# *In situ* determination of Si, N, and P utilization by the demosponge *Tethya citrina*: A benthic-chamber approach

**DOI:** 10.1371/journal.pone.0218787

**Published:** 2019-07-08

**Authors:** María López-Acosta, Aude Leynaert, Laurent Chavaud, Erwan Amice, Isabelle Bihannic, Thierry Le Bec, Manuel Maldonado

**Affiliations:** 1 Department of Marine Ecology, Centro de Estudios Avanzados de Blanes (CEAB-CSIC), Blanes, Girona, Spain; 2 Univ Brest, CNRS, LEMAR, Plouzané, France; 3 Institut Universitaire Européen de la Mer, Université de Bretagne Occidentale, Brest, France; CNRS-UPS, FRANCE

## Abstract

Sponges consume dissolved silicon (DSi) to build their skeletons. Few studies have attempted to quantify DSi utilization by these organisms and all available determinations come from laboratory measurements. Here we measured DSi consumption rates of the sponge *Tethya citrina* in its natural habitat, conducting 24h incubations in benthic chambers. Sponges consumed DSi at an average rate of 0.046 ± 0.018 μmol h^-1^ mL^-1^ when DSi availability in its habitat was 8.3 ± 1.8 μM. Such DSi consumption rates significantly matched the values predicted by a kinetic model elsewhere developed previously for this species through laboratory incubations. These results support the use of laboratory incubations as a suitable approach to learn about DSi consumption. During the field incubations, utilization of other dissolved inorganic nutrients by this low-microbial-abundance (LMA) sponge was also measured. The sponges were net sources of ammonium (-0.043 ± 0.031 μmol h^-1^ mL^-1^), nitrate (-0.063 ± 0.031 μmol h^-1^ mL^-1^), nitrite (-0.007 ± 0.003 μmol h^-1^ mL^-1^), and phosphate (-0.004 ± 0.005 μmol h^-1^ mL^-1^), in agreement with the general pattern in other LMA species. The detected effluxes were among the lowest reported for sponges, which agreed with the low respiration rates characterizing this species (0.35 ± 0.11 μmol-O_2_ h^-1^ mL^-1^). Despite relatively low flux, the dense population of *T*. *citrina* modifies the availability of dissolved inorganic nutrients in the demersal water of its habitat, contributing up to 14% of nitrate and nitrite stocks. Through these effects, the bottom layer contacting the benthic communities where siliceous LMA sponges abound can be partially depleted in DSi, but can benefit from inputs of N and P dissolved inorganic nutrients that are critical to primary producers.

## Introduction

Major dissolved inorganic nutrients (i.e., silicate, ammonium, nitrate, nitrite, and phosphate) are far from being at saturating concentrations in the photic ocean [1–3]. Although geochemical processes may be partially responsible for the existing concentrations, a major role is attributed to processes of consumption and recycling by the microphytoplankton and the bacterioplankton [4–8]. In contrast, the role of benthic marine invertebrates has been less frequently quantified as part of this biological utilization, even when there is evidence that marine sponges are involved in the benthic pelagic coupling of some inorganic nutrients [9–12].

Most sponges consume silicic acid from the seawater to produce their silica skeletons. Silicic acid will be hereafter referred to as DSi, because it dissolved nature. Despite several previous physiological studies [13–18], the rates at which DSi consumption takes place and the factors that control them are still little understood, including potential methodological artifacts. The available direct measurements of DSi consumption by sponges (i.e., values not being estimated from assumed growth rates) have consistently been obtained from hours-to-days incubations in the laboratory [13, 16, 18–22]. Some authors have also attempted to determine rates of DSi consumption in sponges *in situ* by measuring the difference in DSi concentration between the seawater going into the sponge and the seawater going out through the osculum/a (an approach often referred to as “*In-Ex* approach”). However, the flux of water through the sponge is so rapid and retention rate of DSi is so small that the putative consumption rates are at the limit of reliable detectability (e.g., [23–25]). Therefore, the incubation approach to determine DSi consumption is compelled by logistic constraints. It has been assumed that the physiology of the sponges taken to the laboratory will perform identically to that of individuals in their natural habitats. However, such a hypothesis remains poorly tested [26]. Here we have assayed the demosponge *Tethya citrina* by taking the incubation chambers to the natural habitat rather than taking the sponges to the laboratory. We have compared subsequently the *in situ* experimental results with those previously obtained for this same species in laboratory incubations [27]. Thus, this study provides a first test for "laboratory" versus "field" determinations of DSi utilization rates in a shallow-water sponge.

A variety of studies have indicated that, in addition to DSi, sponges can release and/or take up other dissolved inorganic nutrients, such as ammonium, nitrate, nitrite, and phosphate [9, 11, 28–30]. Most of these nutrient influx and outflux are thought to result from a complex combination of physiological processes that include not only the metabolism of the sponge cells but also that of the microbial communities (archaea, bacteria, cyanobacteria, dinoflagellates, etc) that live, sometimes in large quantities, within the sponge body. Because the fluxes of those nutrients through the sponge body often happen at higher rates than DSi flux does, they have been determined efficiently in a few cases by both the *In-Ex* approach [24, 26, 31–33] and laboratory incubations (e.g., [10, 26]). Before this study, only nitrogen (N) fluxes of dissolved inorganic compounds had been measured *in situ* using benthic chambers [34]. Herein, although our primary objective was to measure DSi fluxes, we have taken the opportunity of these *in situ* incubations to examine whether the assayed sponge species is a net source or sink of ammonium, nitrate, nitrite, and phosphate, discussing the results in the frame of the previous knowledge available.

## Materials and methods

### Experimental setup

**Fig 1:**
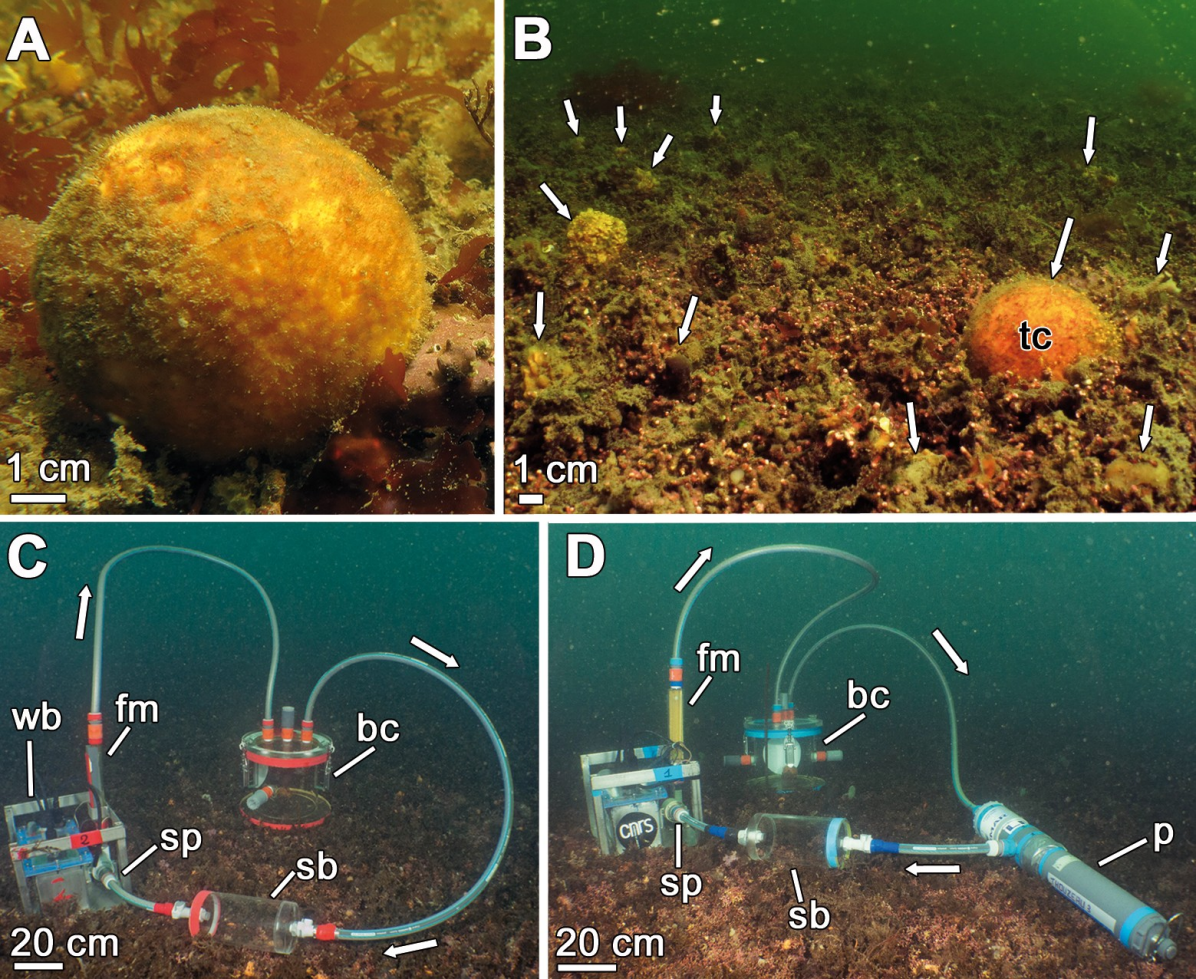
**View of *Tethya citrina* (A-B) and the experimental setup (C-D) at the sponge habitat.** (**A**) Individual of *T*. *citrina* growing at its habitat in the bay of Brest. (**B**) General view of the maërl bed of Lomergat (bay of Brest, France), which hosts a multispecific sponge aggregation, including *T*. *citrina* (tc). White arrows indicate some of the sponges occurring on the maërl. (**C-D**) Each incubation unit consisted of a methyl methacrylate benthic chamber (bc), a submersible pump (sp) connected to a flowmeter (fm) powered by a waterproof battery (wb), and two sampling bottles (sb) hermetically connected to the whole system. Some incubation units (#2, 5, 6, 7, and the control) also incorporated a multiparameter probe (p) that measured oxygen concentration. The seawater flow direction is indicated by white arrows.

We investigated utilization of dissolved inorganic nutrients by the species *Tethya citrina* (Fig 1A and 1B) in the bay of Brest (France). A total of nine incubations were conducted by scuba diving. Eight of them (#1 to #8) were aimed to estimate changes in nutrient concentration in the presence of a sponge individual in the benthic chamber. A ninth incubation chamber served as a control (C), containing only seawater but no sponge. The limited number of incubations and a single control were forced by logistic constraints regarding the number of chambers (n = 4) available for simultaneous replication, security concerns on scuba diving in the bay of Brest, and a variety of technological, economic and logistic issues associated to each chamber deployment. Each incubation unit consisted of a methyl methacrylate chamber, two sampling bottles of 1 L each, and an adjustable submersible pump connected to a flowmeter and powered by a battery (Fig 1C; [35]). Each unit has a capacity of 7.50 ± 0.37 L of seawater and it was hermetically sealed during the incubation. The water was circulated at 2 L min^-1^, similar to the prevailing flow in the natural habitat of the sponge in the bay [36]. Incubations lasted 24h, a period representing a balance between the duration of the battery (about 25h) and a minimum time required to detect reliably changes in nutrient concentration (particularly DSi) due to the sponge activity. In four sponge incubations (#2, 5, 6, and 7) and in the control chamber, a multiparameter probe (YSI 6920) was included in the incubation unit to record dissolved oxygen (% and mg L^-1^), temperature (°C), salinity (ppt), and depth (m) every minute (Fig 1D), with measuring accuracy of 1%. Dissolved oxygen was measured with two purposes: 1) to determine sponge respiration rates and also monitoring whether sponges were physiologically active (i.e., respiring) during the entire incubation period, and 2) to detect whether accidental leakage could have occurred in the chambers during the incubations.

For the incubations, chambers were deployed at 6.5 m depth on the maërl bed of Lomergat (48.290° N, 4.346° W), where the sponges abound (Fig 1B). At the time of the experiments (November 2016), photoperiod was 9:15 light:dark and seawater temperature 11.1 ± 0.5°C, with no substantive changes between day and night. When installing the incubation units on the seafloor, special attention was paid to avoid resuspension of sediments and interstitial nutrients. Individual sponges, each attached to a small piece of maërl, were transferred from the surrounding bottom into the chambers. Once the system was deployed and in place, the pump was turned on while keeping the benthic chamber open, circulating ambient water throughout the entire system during 15 minutes. This ensured that the water to be used during the incubations was the one in the sponge habitat.

Immediately after the incubations, we transported the sampling bottles and the assayed sponges to the laboratory for nutrient analysis and biomass determination. During the trip back to the laboratory, sponges were kept under seawater at all times and nutrients refrigerated. At the laboratory, sponges were first measured in volume (mL) by the water displacement method, and then wet weighted (g), dried at 60°C to constant weight (g), and turned into ash at 540° for 10h. Ash-free dry weight (AFDW; g) was also calculated by subtracting the ash weight from the dry weight. All morphometric parameters measured are included in S1 Table.

### Nutrient analyses

In addition to the utilization of DSi by the sponges, which was our primary objective, we estimated net fluxes of ammonium, nitrate, nitrite, and phosphate over the incubation period. Seawater samples were filtered through a 0.22-μm Whatman-Nucleopore polycarbonate membrane, keeping 50 mL in the fridge prior to DSi determination and freezing 200 mL for subsequent ammonium, nitrate, nitrite, and phosphate determinations. All DSi samples were processed in a single analysis, using a Shimadzu, UV-1700 PharmaSpec UV-VIS spectrophotometer and following the standard colorimetric method [37], with a determination accuracy of 5%. Samples for ammonium, nitrate, nitrite, and phosphate were analyzed using a Technicon Auto-Analyzer (3 HR, SEAL), following the method described by Tréguer and Le Corre [38], with a determination accuracy of 1%.

Nutrient fluxes were calculated as the value of nutrient concentration in the seawater at the beginning of the incubation minus the value after 24h of incubation, being negative values interpreted as nutrient release and positive values as nutrient incorporation by the sponges. Fluxes values were also corrected by the change in concentration occurring in the control unit. Yet we understand that the incubation approach used is more reliable to estimate DSi utilization than fluxes of N and P nutrients, since modification of the concentrations of ammonium, nitrate, nitrite, and phosphate might occur during the 24h incubation, as well as conversion of one N compound into another, as a result of re-filtration, the metabolic activity of the phytoplankton and bacterioplankton in the chambers and, more importantly, the microbiome within the sponge body. Utilization of oxygen by the assayed individuals (#2, 5, 6, 7) was calculated as that of dissolved inorganic nutrients.

Utilization of nutrients and oxygen was normalized by seawater volume in the incubation unit (L), duration of incubation (h), and sponge size (mL). We preferentially used sponge volume for normalization because it facilitates future comparisons without the need of using destructive sampling on the sponge communities. The normalized rates of DSi consumption obtained from the *in situ* incubations were compared with the consumption rates predicted from two kinetic models previously developed by us through laboratory incubations [27]. The two models used for the comparison incorporate the variability in the DSi consumption kinetics in *T*. *citrina* under different seasonal conditions (summer vs autumn).

Finally, we examined the potential pairwise relationships between the fluxes of the different nutrients in the assayed individuals (n = 8), using linear and non-linear regression.

## Results

### Oxygen utilization

**Fig 2:**
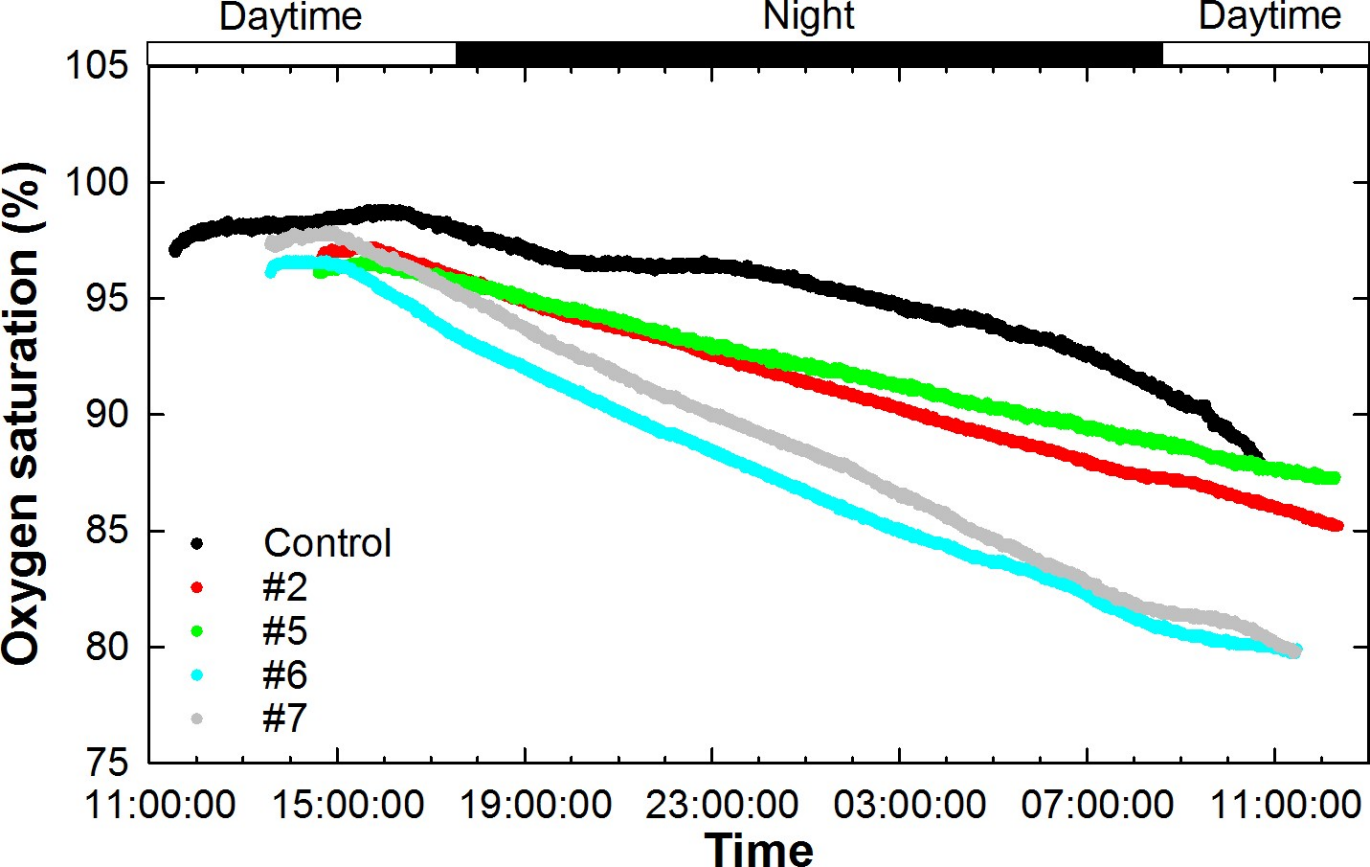
Oxygen saturation profiles during incubations. Dissolved oxygen saturation (%) is plotted against time course during the incubations. Oxygen was measured every minute in five out of nine conducted incubations, considering four incubations with a sponge (#2, 5, 6, and 7) and a control incubation chamber lacking the sponge.

Oxygen measurements revealed that the control unit slightly produced oxygen during daytime due to photosynthesis of the microphytoplankton in suspension (n = 5; -2.21 ± 3.63 μmol O_2_ h^-1^) while consumed oxygen during nighttime because of respiration (n = 18; 7.35 ± 5.54 μmol O_2_ h^-1^). This pattern suggests that the microphytoplankton and the bacterioplankton trapped in the water of the chamber kept active during the incubation (Fig 2). In the incubation units containing sponges, the phytoplankton photosynthesis was initially able to compensate the sponge respiration during daytime (Fig 2; n = 4; -2.39 ± 1.98 μmol O_2_ h^-1^) but soon all units showed a net oxygen consumption (n = 80; 13.73 ± 5.95 μmol O_2_ h^-1^). That oxygen decrease over time was expected. This is so because the incubations took place during nighttime, with only a brief sunset initial period and a brief sunrise final period, which were both characterized by dim light that did not trigger much net photosynthesis and new oxygen production.

The rate of oxygen consumption averaged across all sponges during the corresponding incubation time and after correcting by the control accounted for 0.35 ± 0.11 μmol O_2_ h^-1^ sponge-mL^-1^ (n = 4). The respiration rate measured for the sponges #5, 2, 7, and 6 were, respectively, 0.22, 0.33, 0.38, and 0.48 μmol O_2_ h^-1^ sponge-mL^-1^, that is, relatively similar across individuals. These results also involve that the sponges showed similar levels of physiological activity during the incubations. By expressing the oxygen decrease in the sponge-containing chambers and in the control in mathematical units unrelated to the sponge biomass, that is μmol O_2_ h^-1^, it was determined that the plankton community trapped in the water of the chambers was responsible for about 45 ± 15% of the global oxygen decrease in the chambers_,_ at a rate of 5.77 μmol O_2_ h^-1^. Additionally, because the incubated sponges were attached to a tiny piece of maërl, we examined whether the maërl potential photosynthetic activity during daytime and respiration during nighttime were of significance. If relevant, that stepped signal should have been captured by the oxygen sensors. However, there was no sign of such a stepped pattern, but just a slow progressive oxygen decrease over time (Fig 2), which suggests that the inaccuracy introduced by the maërl presence in our sponge respiration estimates was, if any, negligible.

### Silicate utilization

**Fig 3:**
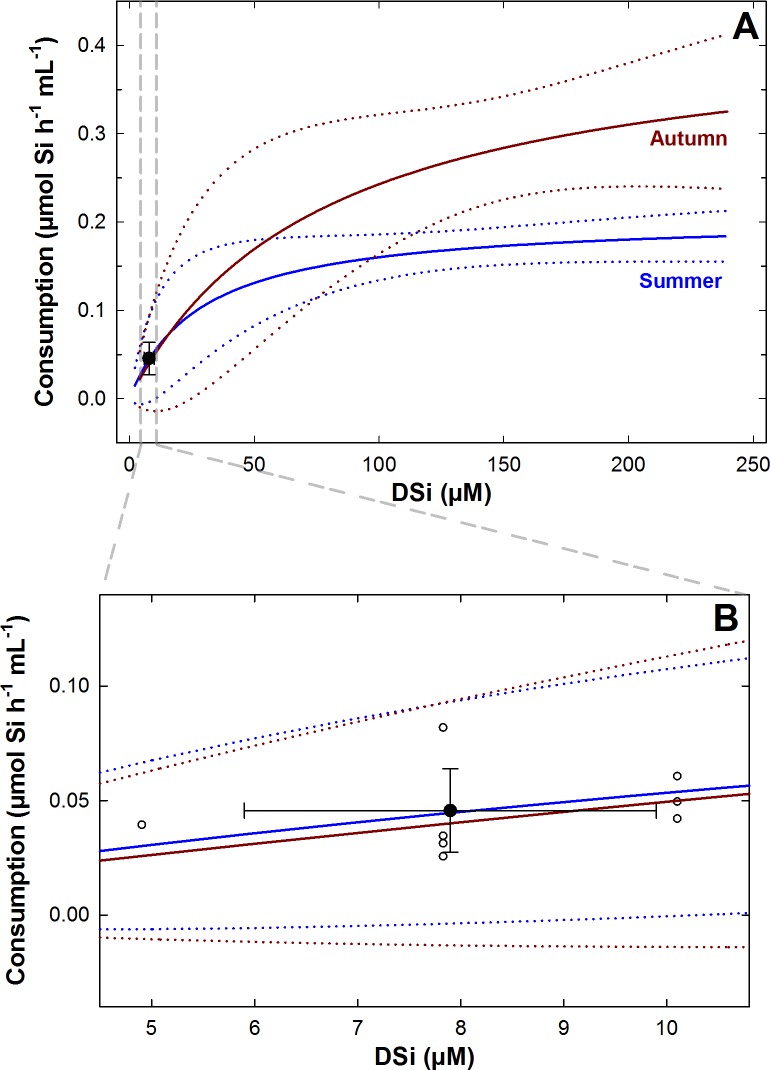
Field versus laboratory rates of DSi consumption in *Tethya citrina*. (**A**) The average (±SD) rate of DSi consumption determined through the *in situ* incubations matched the predictions of the hyperbolic models (solid lines) obtained in the laboratory for *T*. *citrina* for summer and autumn conditions [27]. (**B**) Individual and average (±SD) rates of DSi consumption determined *in situ* (white and black circles, respectively) fell within the 95% confidence band (dotted lines) of both the summer and autumn kinetics.

It took about two weeks (from November 15 to 28, 2016) to complete the 9 incubations in the field. During those two weeks, important river discharges occurred at the bay after heavy rains, causing a slow, progressive increase of the natural DSi concentration, from 4.9 to 10.1 μM (Table 1). The control chamber showed no detectable change in DSi concentration during its incubation (Table 1), indicating that diatoms were scarce or non-active during the period of incubation. This result was expected because diatom abundance is minimal during November in the bay [39, 40]. The individuals of *T*. *citrina* consumed DSi at an average rate of 0.046 ± 0.018 μmol Si h^-1^ mL^-1^ over the incubation period (Table 1). Of note, the increase in DSi in the natural habitat slightly increased the consumption rates through the incubations over November (Table 1). This was expected because DSi consumption in sponges is known to rise with rising DSi availability following a hyperbolic asymptotic function (see [16, 18, 20, 21]). When the consumption rates determined *in situ* in the assayed individuals were compared with the rates predicted by the models elaborated elsewhere through laboratory incubations of *T*. *citrina* [27], their values closely matched (Fig 3). The consumption rate of all incubated individuals fell consistently within the 95% confidence interval of the laboratory kinetic model for DSi consumption in *T*. *citrina*, irrespective of considering the kinetic model developed for either summer or autumn conditions for this species. Also, the average consumption value of the set of assayed individuals fell exactly on top of the summer prediction line and its standard deviation values overlapped the autumn prediction line (Fig 3B). Thus, the agreement between laboratory and field determinations of DSi consumptions rates was nearly total.

**Table 1:** Summary of the nutrient fluxes and its associated parameters. The table contains the reference labels for the 9 conducted incubations, the date of incubation, the size of the incubated sponges, the initial concentration (μM) of the dissolved inorganic nutrients (silicic acid—DSi, ammonium—NH_4_^+^, nitrate—NO_3_^-^, nitrite—NO_2_^-^, phosphate—PO_4_^3-^) at each incubation system and the utilization rate (μmol h^-1^ mL^-1^) normalized by the 24h incubation period and the sponge biomass. Nutrient utilization rates have been corrected by the control effect. The average (±SD) utilization rate by the assayed sponges is indicated in the lower rows of the table.

Code	Date	Size	Initial nutrient level					Nutrient utilization				
			DSi	NH_4_^+^	NO_3_^-^	NO_2_^-^	PO_4_^3-^	DSi	NH_4_^+^	NO_3_^-^	NO_2_^-^	PO_4_^3-^
		(mL)	(μM)					(μmol h^-1^ mL^-1^)				
C	11.15.16	-	4.9	0.8	5.1	0.3	0.5	0.000^a^	0.003^a^	0.026^a^	0.002^a^	0.002^a^
1	11.15.16	20	4.9	0.9	5.0	0.3	0.5	0.039	-0.044	-0.022	-0.012	-0.001
2	11.22.16	18	7.8	1.8	6.1	0.3	0.5	0.035	-0.012	-0.055	-0.004	-0.003
3	11.22.16	17	7.8	1.6	6.0	0.3	0.4	0.031	0.024	-0.038	-0.002	0.002
4	11.22.16	21	7.8	1.5	5.9	0.3	0.4	0.026	-0.066	-0.046	-0.004	0.002
5	11.22.16	11	7.8	1.6	5.2	0.2	0.4	0.082	-0.078	-0.116	-0.008	-0.011
6	11.28.16	19	10.1	1.8	12.3	0.4	0.6	0.049	-0.024	-0.068	-0.010	-0.007
7	11.28.16	27	10.1	1.5	11.1	0.4	0.5	0.061	-0.075	-0.062	-0.006	-0.007
8	11.28.16	13	10.1	1.7	11.5	0.4	0.6	0.042	-0.002	-0.100	-0.009	-0.002
**AVRG**								0.046	-0.043*	-0.063	-0.007	-0.004
**SD**								0.018	0.031*	0.031	0.003	0.005

^a^ Nutrient utilization rates in the control are given in μmol h^-1^.

* Average (±SD) ammonium release rate was calculated from seven sponges that released ammonium, discarding one that took it up.

### N and P utilization

The incubations indicated that all sponges were net sources of nitrate and nitrite. The availability of nitrate during the two weeks of experimentation shifted from 5.0 to 12.3 μM in the natural habitat, while variation was minimal in the case of nitrite (from 0.2 to 0.4 μM). Individual rates of nitrate release ranged from -0.022 to -0.116 μmol h^-1^ mL^-1^, and those of nitrite, from -0.002 to -0.012 μmol h^-1^ mL^-1^. Average rates of nitrate and nitrite production were -0.063 ± 0.031 and -0.007 ± 0.003 μmol h^-1^ mL^-1^, respectively (Table 1). Regarding the ammonium, the natural availability doubled, from 0.8 to 1.8 μM, during the two weeks of experiments. All sponges were net sources of ammonium, except individual #3, which consumed this nutrient at a rate of 0.024 μmol h^-1^ mL^-1^ (Table 1). The rest of the sponges released ammonium at an average rate of -0.043 ± 0.031 μmol h^-1^ mL^-1^.

The natural concentration of phosphate shifted from 0.4 to 0.6 μM over the two weeks of experiments. This is a nutrient for which the sponges showed no consistent pattern in the sign of the flow, with a majority of 6 individuals being net sources (-0.005 ± 0.004 μmol h^-1^ mL^-1^) and a minority of 2 sponges being net sinks (0.002 ± 0.0001 μmol h^-1^ mL^-1^).

**Fig 4:**
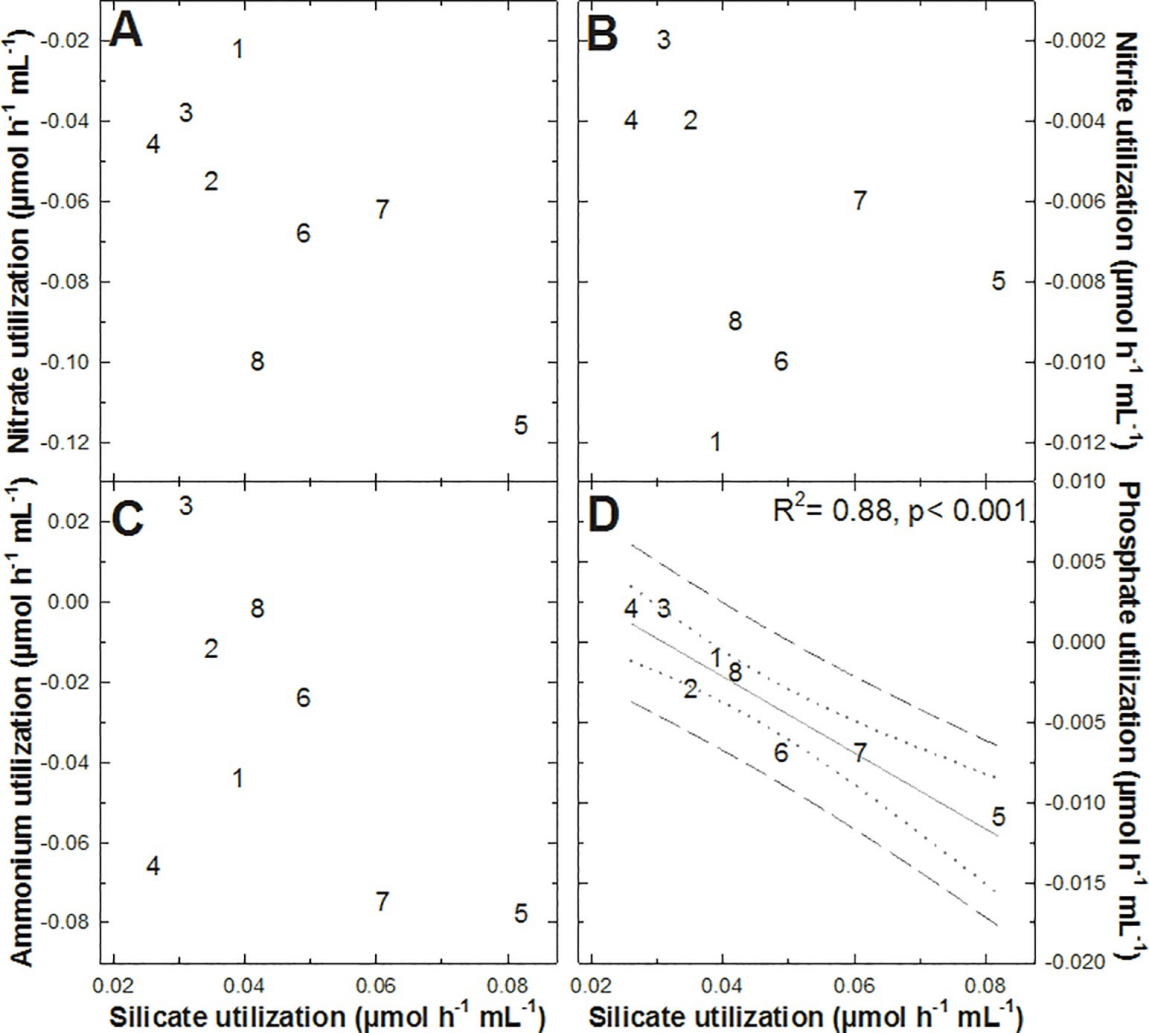
Pairwise relationship between the nutrient fluxes measured in *Tethya citrina*. No statistically significant relationship was found between silicate utilization rates and nitrate (**A**), nitrite (**B**), and ammonium (**C**) utilization rates. In contrast, the consumption of silicate by *T*. *citrina* was linearly related to its release of phosphate (**D**). The rest of possible pairwise relationships between all 4 nutrients was also examined but with non-significant results.

When the rate at which each of these 4 nutrients was released by the sponges was compared to the rate at which DSi was consumed by the sponges (Fig 4), no statistically significant relationship was found, except for one case: the rate of phosphate release increased linearly (p< 0.001, R^2^ = 0.88) with the rising of the silicate consumption (Fig 4D). The two individuals (#3 and 4) showing phosphate consumption were also the ones having the lowest DSi consumption rates. The biological reasons behind the relationship between the phosphate and DSi fluxes remain enigmatic (see Discussion). No statistically significant pairwise coupling between the rest of the nutrients was detected.

## Discussion

This study corroborates that no major departures in DSi utilization rates occur between laboratory and field incubations. The set of *T*. *citrina* individuals incubated in the bay of Brest consumed, on average, 0.046 ± 0.018 μmol Si h^-1^ mL^-1^ (Table 1). Such a field consumption was efficiently predicted by both the summer and autumn kinetic models previously derived from laboratory experiments for this species (Fig 3A). In the range of natural DSi concentration experienced in the field, that is, under a DSi availability lower than about 15 μM, both summer and autumn DSi consumption models for *T*. *citrina* [27] largely match with each other and also with the results of the field incubations (Fig 3B). The excellent agreement between laboratory-based model predictions and field incubations corroborate that DSi consumption rates can reliably be determined through laboratory experiments, what simplifies enormously the logistic approach and may produce more comparable and reproducible results across species and laboratories.

For nutrients others than DSi, some studies have reported that artefactual nutrient utilization rates might result when sponges are studied in the laboratory, out of their natural habitat. For the *in situ* research, the *In-Ex* methods (e.g., [26, 31]) and *in situ* incubations (e.g., [34]) are thought to provide more realistic results, each with its own pros and cons. Unlike for DSi, the fluxes of N and P are due not only to the sponge cell metabolism, but also to that of the microbial communities within the sponges. Those sponge-associated microbiomes have the capacity to readily convert one N nutrient into another, depending on the sponge species and the level of oxygenation of the mesohyl [41]. A complex combination of factors often hinders a straightforward interpretation of measured net rates for N and P nutrients, particularly if incubation periods as long as those required to detect DSi consumption (i.e., 24h or longer; [18]) are used, as it was our case.

To date, most studies on N fluxes have been conducted on shallow-water, high-microbial abundance (HMA) sponges (revised in [11]). These sponges typically remove ammonium from seawater, as a N source for chemo and phototrophic bacteria and energy source for ammonium oxidizing bacteria and archaea. In contrast, they release nitrate and nitrite, as the probable result of the metabolic activity of nitrifying symbiotic bacteria [42]. The species *T*. *citrina* is here believed to be a low microbial-abundance sponge (LMA) which also contains cyanobacteria, since its congener *Tethya aurantium* has been proved to be so [43]. Most LMA sponges are typically net sources of both nitrate and nitrite, and, in many cases, also ammonium [10, 24, 26, 28, 44]. Therefore, regarding the in-going and out-going directions of the various N nutrient fluxes, the results of our incubations (Table 1) strongly agree with the results of previous literature on LMA shallow-water sponges. It is worth mentioning that while nitrite and nitrate flux were highly consistent across the incubated individuals, all our incubated sponges but one (#3) released ammonium. The reasons for singular reversed flux are not clear, but between-individual disparity in the direction of the ammonium flux has also been reported for other sponges [26].

Cyanobacteria and proteobacteria have been reported to dominate the bacterial community in Mediterranean individuals of *T*. *aurantium* [45], a species phylogenetically close to *T*. *citrina*. Whether these microbiome features are also present in the North-Atlantic population of *T*. *citrina* remains unknown. Since sponge cells lack the ability of nitrification (i.e., oxidizing ammonium into nitrite and then nitrate), the released of nitrate and nitrite in *T*. *citrina* is likely, as it has been assumed for other sponges [11], the result of nitrifying bacteria and cyanobacteria inactivating the toxic ammonia excreted by the sponge cells. The released of ammonium in *T*. *citrina* is more difficult to explain. It could be a secondary consequence of the remineralization of organic matter by anaerobic denitrification through associated microbes to produce N_2_ which is subsequently excreted as ammonium ion [46]. It could also result from the nitrogenase activity of N_2_-fixing symbiotic cyanobacteria [47]. In the absence of detailed studies of the microbial metabolism within this specific sponge, the pathways that generate the net efflux of N nutrients from the incubated sponges remain highly speculative.

In the case of P fluxes, the scarce data in the literature suggest that sponges are net sources of this nutrient [10, 24, 28, 48]. Again, our results come into general agreement with those previous studies (Table 1), although two of the incubated sponges took phosphate up at very low rates. The mechanisms behind the release of phosphate and its inverted relationship to DSi consumption (Fig 4D) remain enigmatic. Unpublished data for deep-sea sponges (Maldonado et al, unpublished) do not reveal any relationship between DSi consumption and release of phosphate, so that, it cannot be discarded the possibility that the correlation in *T*. *citrina* is spurious. Of note, the abundance of cyanobacteria in *T*. *citrina* should induce uptake rather than release of phosphate [49]. Given the lack of knowledge regarding the microbiome of *T*. *citrina* and the putative functional integration of the main biochemical pathways, further investigations are necessary to elucidate whether phosphate release is indeed biologically related to DSi consumption.

The composition of the sponge-associated microbiome has a direct impact on N and P fluxes, being likely responsible of the between-species differences reported in nutrient utilization rates [11, 41, 50, 51]. For some species, that composition has also been reported to change between individuals of a same population and between populations of a same species established in different geographical regions and/or environments [52]. Additionally, the disparity of approaches makes the comparison of the rates at which N and P effluxes occur in *T*. *citrina* complicated. The only available study measuring N fluxes in sponges through *in situ* incubations [34] reported an average ammonium efflux of -0.052 ± 0.006 μmol h^-1^ mL^-1^ in two out of three LMA sponges from the Florida Keys (i.e., *Haliclona* sp. and *Halichondria melanodocia*), whereas a third species (*Cinachyrella* sp.) consumed ammonium at an average rate of 0.005 ± 0.002 μmol h^-1^ mL^-1^. Nitrate+nitrite fluxes were only detected in *Cinachyrella* sp. (-0.016 ± 0.007 μmol h^-1^ mL^-1^), being no detectable in *Haliclona* sp. and *H*. *melanodocia*. Rix [53], using 3h laboratory incubations for five LMA sponges from the Red Sea, reported an average efflux rate of -0.079 ± 0.018 μmol h^-1^ mL^-1^ for ammonium, and -0.023 ± 0.014 μmol h^-1^ mL^-1^ for nitrate+nitrite, being all these rates similar to the ones detected herein (-0.043 ± 0.031 and -0.070 ± 0.032 μmol h^-1^ mL^-1^, respectively; Table 1) through 24h field incubations. Jimenez and Ribes [10], who incubated the Mediterranean LMA *Dysidea avara* in the laboratory for 6 h, reported an average release rate of -0.109 μmol NH_4_^+^ h^-1^ mL^-1^, but no detectable flux for nitrate+nitrite. Two studies that used the *In-Ex* approach to estimate ammonium utilization in shallow-water LMA sponges reported average release rates ranging from -0.199 ± 0.012 to -0.260 ± 0.110 μmol h^-1^ mL^-1^ [24, 26]. The *In-Ex* method was also used to estimate rates of nitrate+nitrite release in 5 HMA species from the Florida Keys: *Agelas conifera*, -0.150 ± 0.134 μmol h^-1^ mL^-1^; *Aplysina archeri*, -0.097 ± 0.041 μmol h^-1^ mL^-1^; *Aplysina lacunosa*, -0.116 ± 0.051 μmol h^-1^ mL^-1^; *Ircinia strobilina*, -0.260 ± 0.204 μmol h^-1^ mL^-1^; *Xestospongia muta*, -0.168 ± 0.094 μmol h^-1^ mL^-1^ [26]. All these rates of nitrate+nitrite release were about one order of magnitude larger than the ones measured herein for *T*. *citrina* and also than those in most LMA species from the literature. That same study [26] reported controversial between- and within-species disparity in consumption and release of ammonium, which rendered the resulting average rates unreliable for comparison (see Table 2 in [26]). In summary, it can be concluded that the release rates of N nutrients in *T*. *citrina* are among the lowest known for LMA sponges, suggesting that the combined metabolism of the microbiome and the sponge cells may be integrated through feed-back mechanisms of nutrients and also that the basal metabolism is relatively slow. The latter hypothesis comes into agreement with the relatively low respiration rate measured (see below).

The monitored changes in oxygen concentration during the incubations revealed an average respiration of 0.35 ± 0.11 μmol O_2_ h^-1^ mL^-1^. This rate is in the range of those measured in two other species of the same genus: 0.89 and 0.23 μmol O_2_ h^-1^ mL^-1^ in *T*. *crypta* [54] and *T*. *californiana* [55], respectively. The respiration values known for demosponges range from 0.21 to 24.6 μmol h^-1^ mL^-1^ (reviewed in [56]). Therefore, the respiration values in *Tethya* spp. can be said to fall among the lowest measured for sponges to date. This conclusion is also in congruence with previous studies indicating that respiration in LMA sponges is consistently much lower than in HMA sponges (e.g., [54, 57]).

The species *T*. *citrina* is one of the most frequent species in the rich sponge assemblage of the bay of Brest, but it only represents 15% of the sponge biomass in the maërl community at the bay of Brest [18], being these maërl beds the prevailing habitat in the bay [18, 36]. The biomass of *T*. *citrina* averaged 45.6 ± 76.3 mL m^-2^ [18]. By extrapolating the *in situ* net nutrient fluxes measured in this study, *T*. *citrina* is herein estimated to consume 0.061 ± 0.065 mmol Si m^-2^ d^-1^, and is responsible for releasing -0.057 ± 0.073 mmol NH_4_^+^ m^-2^ d^-1^, -0.094 ± 0.103 mmol NO_3_^-^+NO_2_^-^ m^-2^ d^-1^, and -0.005 ± 0.008 mmol PO_4_^3-^ m^-2^ d^-1^. Such figures are not negligible when compared to the diel net fluxes previously measured for the entire benthic community of the mäerl bed during autumn [58]. Those values, which were initially considering the activity of the microphytobenthos and benthic macroalgae only, disregarded the role of macroinvertebrates such as sponges. The species *T*. *citrina* is here estimated to consume about 5.7% of the DSi released from the bottom of the mäerl bed (-1.07 ± 3.20 mmol m^-2^ d^-1^). The release of N nutrients by *T*. *citrina* contributes to the general efflux of nitrate+nitrite from the bottom (-0.68 ± 2.78 mmol m^-2^ d^-1^) in about 13.8% and about 1.5% of the ammonium (-3.78 ± 2.09 mmol m^-2^ d^-1^). Finally, the phosphate released by the maërl bed (-0.23 ± 0.18 mmol m^-2^ d^-1^) would be increased by the activity of *T*. *citrina* in about 2.1%. Because *T*. *citrina* represents only about 15% of the sponge biomass in this benthic community, an extrapolation to the total sponge biomass, if assuming similar rates of nutrient influx and efflux in the other sponge species, would be of consideration to the bay biogeochemical cycling.

In global terms, our study adds congruence to the growing view that sponges actively participate in the modulation of the local availability of dissolved inorganic nutrients (DSi, ammonium, nitrate, nitrite, and phosphate) in the bottom layers of benthic communities, participating actively in the benthic-pelagic coupling of nutrients. The importance for the ecosystems of that activity will vary depending on the relative abundance of sponges and the species-specific composition.

## Supporting information

S1 Table Morphometric parameters of the assayed sponges of *Tethya citrina*. Summary of volume, wet weight, dry weight, ash weight, and ash-free dry weight (AFDW) data for the set of individuals of *T*. *citrina* (n = 8) used in the *in situ* experimentation.
(PDF)
