# Supplementary material for: In situ determination of Si, N, and P utilization by the demosponge Tethya citrina: A benthic-chamber approach
Source: PLoS One. 2019 Jul 8;14(7):e0218787. doi: 10.1371/journal.pone.0218787 (PMC6613687; doi:10.1371/journal.pone.0218787)
Supplement: S1 Table — Summary of volume, wet weight, dry weight, ash weight, and ash-free dry weight (AFDW) data for the set of individuals of T. citrina (n = 8) used in the in situ experimentation. (PDF) [file pone.0218787.s001.pdf]

S1 Table. Morphometric parameters of the assayed sponges of *Tethya citrina*. Summary of volume, wet weight, dry weight, ash weight, and ash-free dry weight (AFDW) data for the set of individuals of *T. citrina* (n=8) used in the *in situ* experimentation.

Individual	Volume (mL)	Wet Weight (g)	Dry Weight (g)	Ash Weight (g)	AFDW (g)
1	20	19.74	4.173	1.807	2.366
2	18	14.95	2.718	1.456	1.262
3	17	12.64	2.221	1.113	1.108
4	21	18.42	3.334	1.514	1.821
5	11	9.52	1.703	0.825	0.878
6	19	18.71	3.729	1.830	1.899
7	27	26.32	4.618	2.333	2.285
8	13	11.13	1.882	1.017	0.865
